# Development of a questionnaire for assessing the childbirth experience (QACE)

**DOI:** 10.1186/s12884-017-1462-x

**Published:** 2017-08-30

**Authors:** Pierre Carquillat, Françoise Vendittelli, Thomas Perneger, Marie-Julia Guittier

**Affiliations:** 1 University of Applied Sciences and Arts, Western Switzerland, 47 Avenue de Champel, 1206 Geneva, Switzerland; 2 Pôle Femmes et Enfants, Centre Hospitalier Universitaire de Clermont-Ferrand, site Estaing, 1 place Lucie et Raymond Aubrac, 63003 Clermont-Ferrand Cedex 1, France; 3 Clermont Université, Université d’Auvergne, EA 4681, PEPRADE (Périnatalité, grossesse, Environnement, PRAtiques médicales et DEveloppement), Clermont-Ferrand, France; 4 Division of clinical epidemiology, Geneva University Hospitals, and University of Geneva, Geneva, Switzerland; 5 Department of Gynecology and Obstetrics, Geneva University Hospitals, Geneva, Switzerland

**Keywords:** Childbirth experience, Questionnaire, Development, Primiparous Women

## Abstract

**Background:**

Due to its potential impact on women’s psychological health, assessing perceptions of their childbirth experience is important. The aim of this study was to develop a multidimensional self-reporting questionnaire to evaluate the childbirth experience.

**Methods:**

Factors influencing the childbirth experience were identified from a literature review and the results of a previous qualitative study. A total of 25 items were combined from existing instruments or were created de novo. A draft version was pilot tested for face validity with 30 women and submitted for evaluation of its construct validity to 477 primiparous women at one-month post-partum. The recruitment took place in two obstetric clinics from Swiss and French university hospitals. To evaluate the content validity, we compared item responses to general childbirth experience assessments on a numeric, 0 to 10 rating scale. We dichotomized two group assessment scores: “0 to 7” and “8 to 10”. We performed an exploratory factor analysis to identify underlying dimensions.

**Results:**

In total, 291 women completed the questionnaire (response rate = 61%). The responses to 22 items were statistically significant between the 0 to 7 and 8 to 10 groups for the general childbirth experience assessments. An exploratory factor analysis yielded four sub-scales, which were labelled *“relationship with staff”* (4 items), *“emotional status”* (3 items), *“first moments with the new born,”* (3 items) and *“feelings at one month postpartum”* (3 items). All 4 scales had satisfactory internal consistency levels (alpha coefficients from 0.70 to 0.85). The full 25-item version can be used to analyse each item by itself, and the short 4-dimension version can be scored to summarize the general assessment of the childbirth experience.

**Conclusions:**

The Questionnaire for Assessing the Childbirth Experience (QACE) could be useful as a screening instrument to identify women with negative childbirth experiences. It can be used as both a research instrument in its short version and a questionnaire for use in clinical practice in its full version.

**Electronic supplementary material:**

The online version of this article (10.1186/s12884-017-1462-x) contains supplementary material, which is available to authorized users.

## Background

Advancing the understanding of women’s perceptions of their childbirth experience during the perinatal period generates interest from researchers and health practitioners in obstetrics. The importance of assessing the childbirth experience is now known. This significant life event may have an impact on the psychological health of women, including potential benefits or damage [1, 2]. A positive experience can lead to a sense of accomplishment and feelings of self-worth and self-confidence. Negative childbirth experiences can give rise to a feeling of maternal distress, disempowerment, postpartum depression, and even post-traumatic stress disorder [3–7]. These pathological consequences can compromise subsequent pregnancies, mother-infant interactions, and repercussions on the infant’s psychomotor development [8–12]. Studies have also suggested that postnatal depressive symptoms in women could be a predictor of paternal depression and may have negative effects on a couple’s relationship [13–15]. These results indicate the importance of assessing the women’s perceptions of their childbirth experience to identify negative experiences. For this reason, several questionnaires have been developed to describe childbirth experiences. The commonly interrelated factors that were identified as contributing to the construction of the childbirth experience were perceived control, support and relationship with the caregiver, fear, pain and delivery methods [16]. Stevens et al. suggested that perceived control could be an important predictor of childbirth experience [17]. This variable can influence positively or negatively the perception of this event according to women’s personality. Social support conceptualised and measured in different ways has been found to positively influence the childbirth experience [18]. Many studies reported that women with antenatal fear of childbirth may have increased risk of negative experience. For instance, severe fear of childbirth was demonstrated as a risk factor for elective caesarean delivery, especially among multiparous respondents [19, 20]. Childbirth is one of the most painful events that a woman is likely to experience during her life. Many authors explored the relation between pain and satisfaction of birth. Women may hope for a labour and delivery free of pain relief, but many found that they needed or benefited from it [18]. In our last study, we observed that delivery method influenced key factors of the childbirth experience [21]. We did a qualitative study which aimed to determine important elements associated with the first time childbirth experience through interviews, which confirmed the importance of such factors [22]. Participants reported two additional factors to those found in the literature review which are positive or negative emotions and the first moments with the baby. This study also reported that the concordance of expectations of the women prior to delivery with the childbirth experience is a key factor to improve positive women’s views.

A concept analysis of labour and birth experiences by Larkin et al. described the diversity and the complexity of this experience [16]. They defined it as “an individual life event, incorporating interrelated subjective psychological and physiological processes, influenced by social, environmental, organisational and policy contexts”. The attributes identified were: individual, complex, process and life event. Most existing instruments focus on the mother’s experiences, with regard to their satisfaction with the care, rather than the overall perception of the women [23]. The satisfaction concepts that were derived from bio-medical models of childbirth were not clearly defined, and often the distinction between satisfaction with care and satisfaction with the childbirth experience is ambiguous [24, 25]. Another limitation is that the assessed satisfaction with the childbirth experience seemed inappropriate in reflecting the women’s feelings and perceptions about it [26]. Additionally, most questionnaires tend to report homogenous levels of satisfaction, with few women expressing dissatisfaction. So we think it is important to assess women’s positive and negative perceptions of their childbirth experience because satisfaction measures do not capture the multidimensionality of it.

A limited number of instruments have taken into account multiple relevant factors that influence the childbirth experience, and none validated in French language [27–33]. For instance, the Labor Agentry Scale [27] was developed to measure personal control, and the Wijma Delivery Experience Questionnaire was constructed as a uni-dimensional instrument that is used to measure fear of childbirth [34]. To our knowledge, the instruments that are used to assess women’s childbirth experience that cover all of the previous described factors are lacking. The aim of this study was to develop a multidimensional self-reporting questionnaire assessing the women’s perceptions of their childbirth experience. The current study reports on the methodological development of the Questionnaire Assessing the Childbirth Experience (QACE) and provides an analysis of the measurement properties.

## Methods

### Instrument development

The QACE was built in several stages [35, 36]. First, we synthesized the results of previous studies that evaluated factors influencing the childbirth experience. We compared these results with those of our previous qualitative study on this topic [22]. This step allowed us to map a pragmatic combination of items into six main thematic categories. These thematic categories were: expectations (items 9, 15 and 20, Table 2), sensory experiences (items 3 and 16), perceived control (items 8, 10, 11, 12, 14, and 18), relationship with caregivers and the father (items 5, 6, 7, and 13), emotions (items 1, 2, 4, 21, 22, 23, and 24), and the first moments with the baby (items 17, 18, and 19). Several items were taken or adapted from other existing questionnaires [27–33], including the QMAALD (Questionnaire Measuring Attitudes about Labor and Delivery), CEQ (Childbirth experience questionnaire), LAS (Labour Agentry Scale) and PCQ (Pregnancy and Childbirth Questionnaire). Additional items (3, 16, 17, 18, 19, 20, 22, 23, 24, and 25) were created de novo. Items 1 to 19 and 25 measure causal indicators that potentially influence the childbirth experience. Items 20 to 24 measure the consequences of the childbirth experience. They were drafted to cover the identified areas and formulated as positive and negative statements. The response format was a 4-point Likert scale, ranging from *“Totally”*, *“In part”*, *“not so much”*, to *“not at all”*. This format was used to prevent a noncommittal response set. For six items (10 to 16), we distinguished two periods for the response, which included ‘during labour’ or ‘during delivery’. We used a 0 to 10 numeric rating scale (NRS) for two items to assess pain recall (0 = no pain and 10 = excruciating pain) and to provide a general self-assessment of the childbirth experience (0 = very bad and 10 = very good). One additional “item 25”, not included in the QACE, measures the representation of an ideal childbirth: *“According to you, an ideal birth is …”.* They were assessed with six propositions (Vaginal birth, Spontaneous labor, No pain, Caesarean, With the professional of my choice, and Schedule delivery) that the respondents were asked to rank from most important (n°1) to least important (n°6).

In the second stage, we submitted the draft version to a panel of five experts, one with questionnaire expertise and four with childbirth expertise as clinical and academic midwives. We asked them to evaluate the relevance, content coverage, and comprehensiveness of the items. Their comments helped refine the questionnaire for the pre-test.

In the third stage, the QACE was pilot tested for face validity (e.g., comprehension and relevance) among 30 primiparous women [37]. We used two methods for this face-to-face test, the “think aloud” and *“cognitive interviewing”*, methods to obtain feedback from the women [38]. The questionnaire draft was regularly revised based on the women’s comments. Finally, our questionnaire measured six previously described domains of interest through the 25 items in total.

The fourth stage was the evaluation of the measurement properties of the instrument.

### Study sample and data collection

The recruitment was carried out at the University Hospitals of Geneva (Switzerland) and the University Hospital of Clermont-Ferrand (France) maternity wards by four research midwives. Women were recruited during their stay in the maternity ward and asked if they agreed to participate. The inclusion criteria were: speak and read French, primiparous, singleton foetus, gestational age greater than 37 weeks and the new born was not separated from his mother for medical reasons during the maternity stay. We excluded multiparous subjects because, in general, they were more satisfied in their childbirth experiences than primiparous subjects [39]. We can explain this phenomenon by some obstetric factors, including: shorter labor, fewer interventions, and more realistic expectations according to their previous childbirth experiences. Additionally, according to their predisposition to dissatisfaction, the primiparous subjects represented a population of choice to explore factors associated with the childbirth experience. We preferentially sent the questionnaire by e-mail but the option of sending by post was given to participants. The questionnaire was sent to participants at four weeks post-partum. Two recalls if necessary were done up to six weeks post-partum. The online questionnaire procedure was easier for gathering data, had minimal cost, decreased the possibility of data errors with responses automatically stored in a database, and could ensure women’s privacy when answering potentially sensitive questions about perceived partner support during childbirth. The choice of the best time to collect the data regarding the childbirth experience was based on the results and recommendations from previous studies [2, 39–44]. The purpose was threefold: to avoid the initial period of euphoria or denial that can characterize the early postpartum period, to minimize socially desirable responses by completing the questionnaire without the proximity of caregivers, and to be not so far away from the childbirth experience to keep relatively fresh memories.

### Sample size

According to Rouquette & Falissard [45], as a rule of thumb, a minimum of 300 subjects is acceptable to reveal the factor structure. We addressed the QACE to 477 women to anticipate a response rate between 50 and 70%.

### Statistical analyses

We examined the distributions of all of the items to identify any missing responses or the ceiling and floor effects, e.g., the proportions of the most favourable ratings (*“totally”* for positively worded items, *“not at all”* for negatively worded items).

Then, we sought to verify whether the structure of the instrument matched the initial 6 thematic dimensions, using confirmatory factor analysis (CFA). However, the full hypothesized structure could not be confirmed by CFA due to lack of convergence, and a model limited to 20 items and 4 hypothesized dimensions yielded poor goodness-of-fit statistics: root mean squared error of approximation 0.108 (desirable value <0.06, comparative fit index 0.74 (desirable value >0.95), Tucker-Lewis index 0.70 (desirable value >0.95), and standardized root mean squared residual 0.096 (desirable value <0.08) [46]. Upon reviewing the instrument, we realized that while the items in a given thematic dimension were conceptually related, they did not share a common cause (or latent variable), as should be the case for a psychometric instrument, and this explained the low correlations within a thematic dimension. We determined that each item was clinically relevant in its own right and should be reported separately. Furthermore, we then used exploratory factor analysis (EFA) to identify thematically coherent sets of items that would lend themselves to summative scoring, to facilitate reporting (we used 17 items from Table 2, numbered 1–9 and 17–24, excluding the paired items 10–16). We retained factors with an eigenvalue >1, and applied the varimax rotation to facilitate the interpretation of the loadings. At that stage, we also eliminated items that had unclear loading patterns (see Results). We obtained Cronbach alpha coefficients for the scales obtained by EFA.

To measure the construct validity of our instrument, we compared the responses to items in the self-assessment of the general experience of childbirth that were rated on a 0–10 numerical scale. The descriptive analysis of the response distribution for these items suggested a break point at 8. This NRS scores were transformed to categorical values to compare the following two groups: *“NRS scores of 0 to 7”* versus *“NRS scores of 8 to 10”*. Moreover, the 50th percentile of the scores was eight, which allowed us to compare groups of fairly equal size. As the goal for caregivers is for women to have the best childbirth experience possible, we dichotomized the four response modalities to *“totally”* versus *“in part, not so much, and not at all”* or *“not at all”* versus *“not so much, in part, and totally”*, according to the expected positive or negative responses to each item. The proportions were compared between groups, and the differences were tested using the Fisher exact test.

To analyse the score data, we coded the answer format of the 4-point Likert scale, as follows: 1 (totally), 2 (in part), 3 (not so much), and 4 (not at all). The ratings of negatively worded statements were reverse-scored so that the higher scores more reflected a negative childbirth experiences. The means, with their standard deviations, were calculated for continuous variables, and the statistical significance of differences between groups was tested with Student’s t-test.

Statistical analyses were performed using SPSS 22.0 for Windows (SPSS Inc., Chicago, IL, USA), and Stata, version 13.1 (StataCorp, College Station, TX, USA).

## Results

We sent the questionnaire to 477 women between June and December 2014. Seventy-nine per cent of the participants were recruited in Switzerland, and 96% of questionnaires were sent by email. We received 291 completed or quasi-completed questionnaires (61% response rate).

The characteristics of the study population are reported in Table 1. Most of the participants were Swiss or European (86%), native French speakers (83%), and had a secondary to university level of education (97%). Thirty percent had a caesarean section. Among women who had a vaginal delivery 83% had an epidural analgesia.

**Table 1:** General and obstetrical characteristics of the study population

Variables	Values for the study population	Expected values^b^ *N* = 1786 primiparas
Age (y), mean (SD) Range (*N* = 256)	30.85 (4.68), 19–43	Not Avalaible (﻿NA)﻿
Native country (*N* = 271)		
Swiss *n* (%)	98 (36.2)	NA
European^a^ *n* (%)	135 (49.8)	NA
Others than European *n* (%)	38 (14)	NA
Native language (*N* = 270)		
French n (%)	224 (83)	NA
Other than French *n* (%)	46 (17)	NA
Highest level of education (*N* = 270)		
Compulsory	7 (2.6)	NA
Secondary	123 (45.5)	NA
University/HES	140 (51.9)	NA
Mode of delivery *n* (%)		
Spontaneous vaginal delivery	150 (51.5)	853 (47.8)
Instrumental vaginal delivery	55 (18.9)	428 (24)
Elective caesarean	20 (6.8)	115 (6.4)
Emergency caesarean	67 (23)	390 (21.8)
Epidural analgesia if vaginal delivery	170/205 (83)	1108/1280 (86.6)

^a^All others European countries except Switzerland

^b^Statistics 2015 from Geneva University Hospitals (1′786 primiparas at term)

The factor analysis displayed a poor fit of the theoretical model: the confirmatory factor analysis failed to converge (as described in Methods), and the exploratory factor analysis identified multiple mismatches between the high loading factors and anticipated item groupings. Nevertheless, we sought to identify correlated and thematically related items that could by summarized by a summative score, using exploratory factory analysis in a data-driven approach. We started with 17 items (Table 2, excluding paired items 10–16), and finally retained 13 items grouped into 4 dimensions for a short-form of the questionnaire (Table 3). We excluded 4 items from the final EFA, because they could not be naturally classified into one of the 4 dimensions and displayed an unclear loading pattern (items 3, 9, 20, and 24).

**Table 2:** Comparison of childbirth experience assessments with NRS^a^

Generally…	NRS_Experience 0–7	NRS_Experience 8–10	*P* value (Fischer)
1. I felt worried^c^	12/126 (9.5)	32/165 (19.4)	0.02
2. I felt secure^b^	67/126 (53.2)	120/165 (72.7)	0.001
3. I felt strange sensations^c^	19/126 (15.1)	43/165 (26.1)	0.03
4. I felt confident^b^	37/126 (29.4)	73/165 (44.2)	0.011
5. The staff understood and fulfilled my wishes in a satisfactory manner^b^	71/126 (56.3)	136/165 (82.4)	<0.001
6. I felt emotionally supported by the staff who took care of me^b^	63/126 (50)	131/165 (79.4)	<0.001
7. The staff kept me informed of what was happening^b^	73/126 (57.9)	129/165 (78.2)	<0.001
8. I felt I could express myself and give my opinion about decisions about me^b^	55/126 (43.7)	127/165 (77)	<0.001
9. I am satisfied with the way the events unfolded^b^	30/126 (23.8)	123/165 (74.5)	<0.001
10. I managed to successfully use relaxation techniques to help me during womb contractions^b^			
*a. During labour*	26/105 (24.8)	52/151 (34.4)	0.1
*b. During delivery*	16/99 (16.2)	52/144 (36.1)	0.001
11. I managed to successfully move or choose posture freely^b^			
*a. During labour*	40/104 (38.5)	96/149 (64.4)	<0.001
*b. During delivery*	12/97 (12.4)	56/143 (39.2)	<0.001
12. I seemed to lose my head^c^			
*a. During labour*	20/106 (18.9)	67/154 (43.5)	<0.001
*b. During delivery*	24/107 (22.4)	73/154 (47.4)	<0.001
13. The support of my partner helped me^a^			
*a. During labour*	93/109 (85.3)	122/148 (82.4)	0.610
*b. During delivery*	91/107 (85)	127/150 (84.7)	1
14. My pain was relieved when I asked^a^			
*a. During labour*	62/105 (59)	100/142 (70.4)	0.078
*b. During delivery*	55/102 (53.9)	112/140 (80)	<0.001
15. Every event unfolded as I had imagined^a^			
*a. During labour*	6/107 (5.6)	38/153 (24.8)	<0.001
*b. During delivery*	8/113 (7.1)	56/155 (36.1)	<0.001
16. On the numeric rating scale for pain assessment (ranging from 0 to 10), how did you feel pain?^d^			
*a. During labour*	97/117 (82.9)	121/148 (81.8)	0.87
*b. During delivery*	45/120 (37.5)	36/156 (23.1)	0.01
Immediately after childbirth…	NRS_Experience 0–7	NRS_Experience 8–10	*P* value (Fischer)
17. I was able to see my baby for the first time in a satisfactory manner^b^	76/126 (60.3)	126/162 (77.8)	0.002
18. I held my baby for the first time when I felt like it^b^	67/126 (53.2)	125/162 (77.2)	<0.001
19. The first moments with my baby corresponded with what I had imagined prior to giving birth^b^	44/126 (34.9)	98/161 (60.9)	<0.001
Currently…	NRS_Experience 0–7	NRS_Experience 8–10	*P* value (Fischer)
20. I understood everything that happened during childbirth^b^	63/126 (50)	116/157 (73.9)	<0.001
21. I am proud of myself^b^	78/126 (61.9)	137/157 (87.3)	<0.001
22. I feel regret^c^	41/126 (32.5)	102/157 (65)	<0.001
23. I have a feeling of failure^c^	78/126 (61.9)	129/157 (82.2)	<0.001
24. Imagine a subsequent delivery scares me^c^	28/126 (22.2)	82/157 (52.2)	<0.001

^a^Numeric Rating Scale (NRS) 0 to 10 (0 = very bad experience, 10 = very good experience)

^b^Dichotomisation “totally” versus “in part, not so much, not at all”

^c^Dichotomisation “not at all” versus “not so much, in part, totally”

^d^Dichotomisation “VAS for pain 0 à 5” versus “VAS for pain 6 to 10”. We reported 6 to 10

**Table 3:** Factor loading^a^ from the exploratory factor analysis (varimax rotation)

Items	Relationship with staff	Emotional status	First moments with the new born	Feelings at 1 month postpartum
Staff understood and fulfilled my wishes in a satisfactory manner	0.79			
I felt emotionally supported by the staff who took care of me	0.81			
The staff kept me informed of what was happening	0.81			
I felt I could express myself and give my opinion about decisions about me	0.71			
**Cronbach’s Alpha**	**0.81**			
I felt worried		0.79		
I felt confident		0.78		
I felt secure	0.33	0.67		
**Cronbach’s Alpha**		**0.70**		
I was able to see my baby for the first time in a satisfactory manner			0.86	
I held my baby for the first time when I felt like it			0.89	
First moments with my baby corresponded what I had imagined prior to giving birth			0.78	
**Cronbach’s Alpha**			**0.84**	
I am proud of myself				0.71
I feel regret				0.62
I have a feeling of failure			0.31	0.70
**Cronbach’s Alpha**				**0.73**

^a^Only factor loading greater than 0.30 are reported in the table

We computed the dimension scores by averaging the corresponding items if at least 2 items were non-missing and after inversion of the negatively formulated items. The first scale included 4 items that rated interactions and relationships with the staff (items 5, 6, 7, and 8), the second grouped 3 items that rated emotional status (items 1, 2, and 4), the third grouped 3 items that rated the first moment and interaction with the new born (items 17, 18, 19), and the fourth grouped 3 items that rated the feelings at one month after giving birth (items 21, 22, 23).

The Cronbach’s Alpha coefficients for these scores were 0.85, 0.70, 0.84 and 0.72, respectively (Table 3). The differences in the four subscale score between the 0–7 versus 8–10 NRS experiences were significantly different between the *“NRS zero to seven”* group for general self-assessment childbirth experiences compared with the *“NRS eight to ten”* group, which had four item groups that were scored higher (Table 4). The scales were weakly to moderately correlated (Table 5).

**Table 4:** Differences in the subscales score between NRS_Experience 0–7 versus 8–10

Dimensions	NRS* _Experience 0–7 *N* = 126	NRS*_Experience 8–10 *N* = 165	t-test
Relationship with staff mean, ﻿subscale score**﻿ (SD)	1.65 (0.65)	1.26 (0.44)	< 0.001
Emotional status mean, ﻿subscale score**﻿ (SD)	2.04 (0.62)	1.78 (0.58)	< 0.001
First moments with the new born mean, ﻿subscale score**﻿ (SD)	2.03 (1.03)	1.52 (0.82)	< 0.001
Feelings at 1 month postpartum mean, ﻿subscale score**﻿ (SD)	1.82 (0.79)	1.34 (0.50)	< 0.001

*NRS = Numeric Rating Scale of general self-assessment experience (high score associated with positive experience)

**﻿Higher score﻿﻿ reflected a negative childbirth experiences

**Table 5:** Pearson correlation coefficients between the four summary scales

	Emotional status	First moments with newborn	Feelings at 1 month post-partum
Relationship with staff	0.37	0.28	0.47
Emotional status		0.20	0.27
First moments with newborn			0.43

We therefore propose 2 possible uses for the instrument:

- Use of each of the 24 items in their own right if the emphasis was on the specific content of each item (Table 2). The general self-assessment childbirth experience, as reported with the NRS, showed that 43% (*n* = 126) of the women estimated that their experience was between 0 to 7 compared with 57% (*n* = 165) who estimated that their experience was between 8 to 10. The difference between the positive and negative perception groups was statistically significant for all items except 2 (the support of the partner and the pain assessment during labour). Complete version of the QACE in English language is available in Additional file 1 and in French language in Additional file 3.
- When summary descriptions of the mother’s experience are needed, we suggest the use of the 4 summative scales, based on 13 items. This simplified tool may be particularly useful for research or monitoring. Short version of the QACE in English language is available in Additional file 2 and in French language in Additional file 4.

The women’s responses to their representations of an ideal birth (item 25) that ranked first and second, in quasi equality, were *“Spontaneous labour”* 114/283 (40.3%) and *“Vaginal birth”* 112/283 (39.6%). *“No pain”* 107/283 (37.8%) was third, “with the professional of my choice” 125/283 (44.5%) was fourth, “scheduled” 126/283 (45.3%) was fifth, and “by caesarean” 148/283 (53%) was sixth.

## Discussion

In this study, we developed a self-reporting questionnaire in French to assess the childbirth experiences of first-time mothers. Our findings suggest that the QACE could be used as an instrument to identify women with negative experiences, to perform quality of care monitoring in birthplaces, and to assess childbirth experiences by researchers. The full version (25 items) is an index that is used to analyse each item by itself as a *“clinimetric scale”* [47]. The short version contained 13 items, with a total of 4 sub-scales to measure the general assessment of the childbirth experience with scores per dimension.

### Strengths

This QACE was developed following a rigorous methodological protocol [35, 36]. The initial list of domains and corresponding pool of items were derived from a literature review on childbirth experiences and the existing measurement instruments for them. We created new items to specifically evaluate representations of an ideal childbirth, comprehension of events during childbirth, and first moments with the new born according to the results of our previous qualitative study on the childbirth experience [22]. The generation of these items ensured that the core determinants of the delivery experience were not missed. Several successive revisions were performed according to opinions from a panel of clinical and methodological experts and a pilot-test with 30 women to maximize the content validity. The *“support of the partner”* and *“feeling of pain during labour”* items were not consistent with what was expected. This was surprising and different from factors that were traditionally associated with the delivery experience [48–50]. In our study, more than 76% of the participants reported to be totally supported by their partner; however, we did not observe a positive association with the NRS in their self-assessments of the delivery experience. For the feeling of pain, 83% of the participants had an epidural analgesia. This could explain the lack of an association between pain and the NRS of their self-assessment of the delivery experience. Nevertheless, we decided to keep these items in the full version because they can be helpful in another context of evaluation.

### Limits

The exploratory factor analysis did not identify the six thematic categories as expected; however, the grouping of the extracted items made sense and could be labelled for the initially described thematic categories, except for *“sensory perceptions”* (items 3, 16) and *“feeling of control”* (items 8, 10, 11, 12, 14, 18). The lack of consensus about the definition of some thematic categories reported in different instruments to assess childbirth experience could explain the heterogeneity of the results. For instance, in many research studies, the control concept was insufficiently defined [16]. Consequently, items related to the feeling of control are often included in other concepts in questionnaires. For instance, in the Childbirth Experience Questionnaire [32], the dimension labelled *“Own capacities”* included items regarding experienced emotions, sense of control and experienced labour pain. *“Feeling of control”* is traditionally described as very important for the childbirth experience [44, 51–53]. Nevertheless, the feeling of control is very subjective. This is a complex phenomenon and includes internal and external dimensions. Additionally, we suggest weighing the impact of the feeling of control during the childbirth experience with regards to the two psychological profiles described by Green et al. [39], which include a woman’s desire to *“participate in decisions”* or conversely to *“surrender to the medical world”*. This point of view is also supported by Stevens et al. (2012) [17]. Moreover, this sense of control is not static during labour and birth, as it evolves according to the sequence of events and the responses to each. A unidimensional tool regarding this exists [27] and could be submitted in parallel to the QACE if researchers want to measure this latent variable. Another example of the heterogeneity of the thematic categories is given by Bryanton et al. [54]. They explained that fear was not a predictor in any of the studies that examined several predictors simultaneously. Fear was represented in other studies by variables such as anxiety, loss of control, and worry about the baby.

A woman’s mood on the day she answers the questionnaire can change the perception of the childbirth experience [6, 55]. During this period, women can feel weary and tired and postnatal depression can appear [56]. This is a scientific bias for the responses to any item that questions memories of emotions. Additionally, 186 (39%) women declined to participate, and this was a potential selection bias that may have affected the final assessment.

The four dimension-specific scores were obtained through a data-driven process and were not based on an a priori measurement model. Therefore, these dimensions may be over-fitted in our sample. A verification of the relevance of these dimensions in other samples is warranted.

### A clinimetric approach rather a psychometric statistical approach

This QACE was developed primarily as a clinical instrument in which each specific item provided information about the women’s childbirth experiences. Feinstein describe it as *“clinimetrics scales”* [47] and Streiner described it as *“indexe”* [57]. The full version of the QACE did not include a psychometric scale composed of theoretically correlated items that would be caused by a hypothesized latent variable. This QACE is a screening instrument that is derived from items that have been found to empirically correlate with the childbirth experience. According to Streiner [57], it is important to differentiate the two test construction models to reflect the fact that different statistical approaches are used with them. Psychometrics test models are not appropriate for indexes for the QACE, although they remain the most widely used method to assess questionnaire validities. As stated by Juniper et al. [58], it *“can lead to situations in which either a scale is wrongly dismissed for not being reliable or the indexes are unfairly criticized for not yielding useful results”*.

The short version was a thematic grouping that could facilitate the interpretation the women’s responses by summarizing the general childbirth experiences with scores for four key dimensions. Higher scores reflected more negative childbirth experiences. We suggest that practitioners or researchers add some items to the short version from the full version, according to their specific domains of interest.

## Conclusions

Assessing the childbirth experience is important because a negative experience could be harmful for a woman’s health as well as her relationship with her partner and infant. The QACE is a multidimensional questionnaire that was developed to identify women with negative experiences. It can be used as both a research instrument in its short version and a questionnaire for use in clinical practice in its full version. Future research studies are needed to evaluate the predictive value of the QACE for the detection of multiple consequences due to negative childbirth experiences, particularly the occurrence of postnatal depression. As well, studies with diverse populations will help to improve the reliability and validity of the QACE.

## Additional files

Additional file 1: English language versions of the QACE (complete version). (DOC 107 kb)

Additional file 2: English language versions of the QACE (short version). (DOC 70 kb)

Additional file 3: French language of the QACE (complete version). (DOC 102 kb)

Additional file 4: French language of the QACE (short version). (DOC 68 kb)

## Abbreviations

CEQ: Childbirth Experience Questionnaire
CFA: Confirmatory Factor Analysis
EFA: Exploratory Factor Analysis
LAS: Labour Agentry Scale
NRS: Numeric Rating Scale
PCQ: Pregnancy and Childbirth Questionnaire
QACE: Questionnaire ﻿for Assessing the Childbirth Experience
QMAALD: Questionnaire Measuring Attitudes About Labor and Delivery
