# Supplementary material for: Development of a questionnaire for assessing the childbirth experience (QACE)
Source: BMC Pregnancy Childbirth. 2017 Aug 30;17:279. doi: 10.1186/s12884-017-1462-x (PMC5577741; doi:10.1186/s12884-017-1462-x)
Supplement: Supplementary file 1 — English language versions of the QACE (complete version). (DOC 107 kb) [file 12884_2017_1462_MOESM1_ESM.doc]

Additional file 1: English language of the QACE (complete version)

**QACE : Questionnaire for Assessing the Childbirth Experience, vaginal birth or** **caesarean**

# Generally…

|  | | **Totally** | **In part** | **Not so much** | **Not at all** |
| --- | --- | --- | --- | --- | --- |
| **1.** | **I felt worried** | 🞏 | 🞏 | 🞏 | 🞏 |
| **2.** | **I felt secure** | 🞏 | 🞏 | 🞏 | 🞏 |
| **3.** | **I felt strange sensations** | 🞏 | 🞏 | 🞏 | 🞏 |
| **4.** | **I felt confident** | 🞏 | 🞏 | 🞏 | 🞏 |
| **5.** | **The staff understood and fulfilled my wishes in a satisfactory manner** | 🞏 | 🞏 | 🞏 | 🞏 |
| **6.** | **I felt emotionally supported by the staff who took care of me** | 🞏 | 🞏 | 🞏 | 🞏 |
| **7.** | **The staff kept me informed of what was happening** | 🞏 | 🞏 | 🞏 | 🞏 |
| **8.** | **I felt I could express myself and give my opinion about decisions about me** | 🞏 | 🞏 | 🞏 | 🞏 |
| **9.** | **I am satisfied with the way the events unfolded** | 🞏 | 🞏 | 🞏 | 🞏 |

|  | | **During labor**  (From the first contractions to the first expulsive efforts) | | | | | **During delivery**  (From the first expulsive efforts to birth OR during the caesarean section) | | | | |
| --- | --- | --- | --- | --- | --- | --- | --- | --- | --- | --- | --- |
|  |  | *Not concerned | Totally | In part | Not so much | Not at all | *Not concerned | Totally | In part | Not so much | Not at all |
| **10.** | **I managed to successfully use relaxation techniques to help me during womb contractions** | 🞏 | 🞏 | 🞏 | 🞏 | 🞏 | 🞏 | 🞏 | 🞏 | 🞏 | 🞏 |
| **11.** | **I managed to successfully move or choose my posture freely** | 🞏 | 🞏 | 🞏 | 🞏 | 🞏 | 🞏 | 🞏 | 🞏 | 🞏 | 🞏 |
| **12.** | **My pain was relieved when I asked for it to be** | 🞏 | 🞏 | 🞏 | 🞏 | 🞏 | 🞏 | 🞏 | 🞏 | 🞏 | 🞏 |
| **13.** | **Every event unfolded as I had imagined it** | 🞏 | 🞏 | 🞏 | 🞏 | 🞏 | 🞏 | 🞏 | 🞏 | 🞏 | 🞏 |
| **14.** | **I felt like I was losing control** | 🞏 | 🞏 | 🞏 | 🞏 | 🞏 | 🞏 | 🞏 | 🞏 | 🞏 | 🞏 |
| **15.** | **The support of my partner helped me** | 🞏 | 🞏 | 🞏 | 🞏 | 🞏 | 🞏 | 🞏 | 🞏 | 🞏 | 🞏 |
|  |  | My partner was not present during labor 🞏 | | | | | My partner was not present during delivery🞏 | | | | |

* Not concerned = I had a caesarean before having a phase of labor with contractions

1. **On the numeric rating scale for pain assessment, how much did you feel pain?**

(Circle the corresponding number on the two scales below)

1. **During labor:** 🞏 Not concerned (I had a caesarean before having contractions)

| **No pain** | 0 | 1 | 2 | 3 | 4 | 5 | 6 | 7 | 8 | 9 | 10 | **Excruciating pain** |
| --- | --- | --- | --- | --- | --- | --- | --- | --- | --- | --- | --- | --- |

1. **During delivery** (caesarean or vaginal delivery)

| **No pain** | 0 | 1 | 2 | 3 | 4 | 5 | 6 | 7 | 8 | 9 | 10 | **Excruciating pain** |
| --- | --- | --- | --- | --- | --- | --- | --- | --- | --- | --- | --- | --- |

# Immediately after childbirth…

|  | | **Totally** | **In part** | **Not so much** | **Not at all** |
| --- | --- | --- | --- | --- | --- |
| **17.** | **I was able to see my baby for the first time in a satisfactory manner** | 🞏 | 🞏 | 🞏 | 🞏 |
| **18.** | **I held my baby for the first time when I felt like it** | 🞏 | 🞏 | 🞏 | 🞏 |
| **19.** | **The first moments with my baby corresponded with what I had imagined prior to giving birth** | 🞏 | 🞏 | 🞏 | 🞏 |

# Currently…

|  | | **Totally** | **In part** | **Not so much** | **Not at all** |
| --- | --- | --- | --- | --- | --- |
| **20.** | **I understood everything that happened during childbirth t** | 🞏 | 🞏 | 🞏 | 🞏 |
| **21.** | **I am proud of myself** | 🞏 | 🞏 | 🞏 | 🞏 |
| **22.** | **I feel regret** | 🞏 | 🞏 | 🞏 | 🞏 |
| **23.** | **I have a feeling of failure** | 🞏 | 🞏 | 🞏 | 🞏 |
| **24.** | **Imagine a subsequent delivery scares me** | 🞏 | 🞏 | 🞏 | 🞏 |

# According to you, an ideal birth is a birth …

(Number the proposals in order of importance from 1 to 6: 1 = the most important of the 6 answers, 6 = the least important of the 6 answers, please don’t put the same number twice)

|  | **Number from 1 to 6** |
| --- | --- |
| Vaginal birth | **N°** |
| Spontaneous labor | **N°** |
| No pain | **N°** |
| Caesarean | **N°** |
| With the professional of my choice | **N°** |
| Schedule delivery | **N°** |

# If you put aside the emotions about the arrival of your baby, for you as a woman, your experience of childbirth has been ...

(Circle the corresponding number on the scale below)

| **very bad experience** | 0 | 1 | 2 | 3 | 4 | 5 | 6 | 7 | 8 | 9 | 10 | **very good experience** |
| --- | --- | --- | --- | --- | --- | --- | --- | --- | --- | --- | --- | --- |

# Free comments

**Here you can freely share points not mentioned in the questionnaire or those that need to be specified.**

------------------------------------------------------------------------------------------------------------------------------------------------------------------------------------------------------------------------------------------------------------------------------------------------------------------------------------------------------------------------------------------------------------------------------------------
