# Supplementary material for: Development of a questionnaire for assessing the childbirth experience (QACE)
Source: BMC Pregnancy Childbirth. 2017 Aug 30;17:279. doi: 10.1186/s12884-017-1462-x (PMC5577741; doi:10.1186/s12884-017-1462-x)
Supplement: Supplementary file 2 — English language versions of the QACE (short version). (DOC 70 kb) [file 12884_2017_1462_MOESM2_ESM.doc]

Additional file 2: French language of the QACE (short version)

**QACE : Questionnaire for Assessing the Childbirth Experience, vaginal birth or** **caesarean**

# Generally…

|  | | **Totally** | **In part** | **Not so much** | **Not at all** |
| --- | --- | --- | --- | --- | --- |
| **1.** | **I felt worried** | 🞏 | 🞏 | 🞏 | 🞏 |
| **2.** | **I felt secure** | 🞏 | 🞏 | 🞏 | 🞏 |
| **3.** | **I felt confident** | 🞏 | 🞏 | 🞏 | 🞏 |
| **4.** | **The staff understood and fulfilled my wishes in a satisfactory manner** | 🞏 | 🞏 | 🞏 | 🞏 |
| **5.** | **I felt emotionally supported by the staff who took care of me** | 🞏 | 🞏 | 🞏 | 🞏 |
| **6.** | **The staff kept me informed of what was happening** | 🞏 | 🞏 | 🞏 | 🞏 |
| **7.** | **I felt I could express myself and give my opinion about decisions about me** | 🞏 | 🞏 | 🞏 | 🞏 |

# Immediately after childbirth…

|  | | **Totally** | **In part** | **Not so much** | **Not at all** |
| --- | --- | --- | --- | --- | --- |
| **8.** | **I was able to see my baby for the first time in a satisfactory manner** | 🞏 | 🞏 | 🞏 | 🞏 |
| **9.** | **I held my baby for the first time when I felt like it** | 🞏 | 🞏 | 🞏 | 🞏 |
| **10.** | **The first moments with my baby corresponded with what I had imagined prior to giving birth** | 🞏 | 🞏 | 🞏 | 🞏 |

# Currently…

|  | | **Totally** | **In part** | **Not so much** | **Not at all** |
| --- | --- | --- | --- | --- | --- |
| **11.** | **I am proud of myself** | 🞏 | 🞏 | 🞏 | 🞏 |
| **12.** | **I feel regret** | 🞏 | 🞏 | 🞏 | 🞏 |
| **13.** | **I have a feeling of failure** | 🞏 | 🞏 | 🞏 | 🞏 |
