# Supplementary material for: Development of a questionnaire for assessing the childbirth experience (QACE)
Source: BMC Pregnancy Childbirth. 2017 Aug 30;17:279. doi: 10.1186/s12884-017-1462-x (PMC5577741; doi:10.1186/s12884-017-1462-x)
Supplement: Supplementary file 3 — French language of the QACE (complete version). (DOC 102 kb) [file 12884_2017_1462_MOESM3_ESM.doc]

Additional file 3: French language of the QACE (complete version)

**QEVA : Questionnaire d’Evaluation du Vécu de l’Accouchement, par voie-basse ou par césarienne**

**(Version complète)**

# D’une façon générale …

|  | | Tout à fait | En partie | Pas tellement | Pas du tout |
| --- | --- | --- | --- | --- | --- |
| **1.** | **Je me sentais inquiète** | 🞏 | 🞏 | 🞏 | 🞏 |
| **2.** | **Je me sentais en sécurité** | 🞏 | 🞏 | 🞏 | 🞏 |
| **3.** | **J’ai ressenti des sensations bizarres** | 🞏 | 🞏 | 🞏 | 🞏 |
| **4.** | **Je me sentais confiante** | 🞏 | 🞏 | 🞏 | 🞏 |
| **5.** | **L’équipe soignante comprenait et répondait à mes désirs de manière satisfaisante** | 🞏 | 🞏 | 🞏 | 🞏 |
| **6.** | **Je me suis sentie soutenue émotionnellement par les professionnels qui s’occupaient de moi** | 🞏 | 🞏 | 🞏 | 🞏 |
| **7.** | **Les professionnels me tenaient informée de ce qui se passait** | 🞏 | 🞏 | 🞏 | 🞏 |
| **8.** | **Je sentais que je pouvais m’exprimer et donner mon avis à propos des décisions me concernant** | 🞏 | 🞏 | 🞏 | 🞏 |
| **9.** | **Je suis satisfaite de la manière dont les évènements se sont déroulés** | 🞏 | 🞏 | 🞏 | 🞏 |

|  | | **Pendant le travail**  **(des premières contractions jusqu’aux premières poussées)** | | | | | **Pendant l’accouchement**  **(des premières poussées jusqu’à la naissance OU durant la césarienne)** | | | | |
| --- | --- | --- | --- | --- | --- | --- | --- | --- | --- | --- | --- |
|  |  | *Pas concernée | Tout à fait | En  partie | Pas tellement | Pas du tout | *Pas concernée | Tout à fait | En partie | Pas tellement | Pas du tout |
| **10.** | **J’ai réussi à utiliser des méthodes de relaxation pour m’aider lors des contractions** | 🞏 | 🞏 | 🞏 | 🞏 | 🞏 | 🞏 | 🞏 | 🞏 | 🞏 | 🞏 |
| **11.** | **J’ai pu me mouvoir ou choisir librement ma position** | 🞏 | 🞏 | 🞏 | 🞏 | 🞏 | 🞏 | 🞏 | 🞏 | 🞏 | 🞏 |
| **12.** | **On a pu soulager ma douleur au moment où je l’ai demandé** | 🞏 | 🞏 | 🞏 | 🞏 | 🞏 | 🞏 | 🞏 | 🞏 | 🞏 | 🞏 |
| **13.** | **Tout s’est déroulé comme je l’avais imaginé** | 🞏 | 🞏 | 🞏 | 🞏 | 🞏 | 🞏 | 🞏 | 🞏 | 🞏 | 🞏 |
| **14.** | **J’avais l’impression de perdre tous mes moyens** | 🞏 | 🞏 | 🞏 | 🞏 | 🞏 | 🞏 | 🞏 | 🞏 | 🞏 | 🞏 |
| **15.** | **Le soutien de mon partenaire m’a aidé** | 🞏 | 🞏 | 🞏 | 🞏 | 🞏 | 🞏 | 🞏 | 🞏 | 🞏 | 🞏 |
|  |  | **Mon partenaire n’était pas présent**  **pendant le travail** 🞏 | | | | | **Mon partenaire n’était pas présent pour l’accouchement** 🞏 | | | | |

*Pas concernée= j’ai eu une césarienne avant d’avoir une phase de travail avec des contractions

1. **Sur une échelle de 0 à 10, à quel point avez-vous éprouvé de la douleur ?**

**(Entourez le chiffre correspondant sur les deux échelles ci-dessous)**

1. **Durant le travail :** 🞏 Pas concernée

| **Pas de douleur** | 0 | 1 | 2 | 3 | 4 | 5 | 6 | 7 | 8 | 9 | 10 | **Douleur extrême** |
| --- | --- | --- | --- | --- | --- | --- | --- | --- | --- | --- | --- | --- |

1. **Durant l’accouchement (césarienne ou voie-basse)**

| **Pas de douleur** | 0 | 1 | 2 | 3 | 4 | 5 | 6 | 7 | 8 | 9 | 10 | **Douleur extrême** |
| --- | --- | --- | --- | --- | --- | --- | --- | --- | --- | --- | --- | --- |

# Immédiatement après la naissance

|  | | Tout à fait | En partie | Pas tellement | Pas du tout |
| --- | --- | --- | --- | --- | --- |
| **17.** | **J’ai pu découvrir visuellement mon bébé de manière satisfaisante** | 🞏 | 🞏 | 🞏 | 🞏 |
| **18.** | **J’ai eu mon bébé contre moi pour la première fois au moment où j’en ai eu envie** | 🞏 | 🞏 | 🞏 | 🞏 |
| **19.** | **Les premiers instants avec mon bébé correspondaient à ce que j’avais imaginé avant d’accoucher** | 🞏 | 🞏 | 🞏 | 🞏 |

# A ce jour

|  | | Tout à fait | En partie | Pas tellement | Pas du tout |
| --- | --- | --- | --- | --- | --- |
| **20.** | **J’ai compris tout ce qui s’est passé lors de mon accouchement** | 🞏 | 🞏 | 🞏 | 🞏 |
| **21.** | **Je suis fière de moi** | 🞏 | 🞏 | 🞏 | 🞏 |
| **22.** | **J’ai des regrets** | 🞏 | 🞏 | 🞏 | 🞏 |
| **23.** | **J’ai un sentiment d’échec** | 🞏 | 🞏 | 🞏 | 🞏 |
| **24.** | **L’idée d’accoucher une nouvelle fois m’effraie** | 🞏 | 🞏 | 🞏 | 🞏 |

# Selon vous, un accouchement idéal est un accouchement…

**(Numérotez de 1 à 6 les propositions par ordre d’importance : 1= la plus importante des 6 réponses, 6= la moins importante des 6 réponses. Attention de ne pas mettre deux fois le même chiffre s’il-vous-plaît)**

|  | Numérotez de 1 à 6 |
| --- | --- |
| Par voie-basse | **N°** |
| Se déclenchant naturellement | **N°** |
| Sans douleur | **N°** |
| Par césarienne | **N°** |
| Avec le professionnel de mon choix  (sage-femme, médecin) | **N°** |
| A une date programmée | **N°** |

# Si l’on met de côté les émotions relatives à l’arrivée de votre bébé, pour vous en tant que femme, votre vécu de l’accouchement a été…

**(Entourez le chiffre correspondant sur l’échelle ci-dessous)**

| **Très mauvais** | 0 | 1 | 2 | 3 | 4 | 5 | 6 | 7 | 8 | 9 | 10 | **Très bon** |
| --- | --- | --- | --- | --- | --- | --- | --- | --- | --- | --- | --- | --- |

# Commentaires libres

**Ici vous pouvez librement partager les points non mentionnés dans le questionnaire ou ceux qui doivent être précisés. Vous pouvez écrire au dos ou sur une autre feuille si cet espace ne suffit pas**

---------------------------------------------------------------------------------------------------------------------------------------------------------------------------------------------------------------------------------------------------------------------------------------------------------------------------------------------------------------------------------------------------------------------------------------------------------------------------------
