# Supplementary material for: Development of a questionnaire for assessing the childbirth experience (QACE)
Source: BMC Pregnancy Childbirth. 2017 Aug 30;17:279. doi: 10.1186/s12884-017-1462-x (PMC5577741; doi:10.1186/s12884-017-1462-x)
Supplement: Supplementary file 4 — French language of the QACE (short version). (DOC 68 kb) [file 12884_2017_1462_MOESM4_ESM.doc]

Additional file 4: French language of the QACE (short version)

**QEVA : Questionnaire d’Evaluation du Vécu de l’Accouchement, par voie-basse ou par césarienne (Version courte)**

# D’une façon générale …

|  | | Tout à fait | En partie | Pas tellement | Pas du tout |
| --- | --- | --- | --- | --- | --- |
| **1.** | **Je me sentais inquiète** | 🞏 | 🞏 | 🞏 | 🞏 |
| **2.** | **Je me sentais en sécurité** | 🞏 | 🞏 | 🞏 | 🞏 |
| **3.** | **Je me sentais confiante** | 🞏 | 🞏 | 🞏 | 🞏 |
| **4.** | **L’équipe soignante comprenait et répondait à mes désirs de manière satisfaisante** | 🞏 | 🞏 | 🞏 | 🞏 |
| **5.** | **Je me suis sentie soutenue émotionnellement par les professionnels qui s’occupaient de moi** | 🞏 | 🞏 | 🞏 | 🞏 |
| **6.** | **Les professionnels me tenaient informée de ce qui se passait** | 🞏 | 🞏 | 🞏 | 🞏 |
| **7.** | **Je sentais que je pouvais m’exprimer et donner mon avis à propos des décisions me concernant** | 🞏 | 🞏 | 🞏 | 🞏 |

# Immédiatement après la naissance

|  | | Tout à fait | En partie | Pas tellement | Pas du tout |
| --- | --- | --- | --- | --- | --- |
| **8.** | **J’ai pu découvrir visuellement mon bébé de manière satisfaisante** | 🞏 | 🞏 | 🞏 | 🞏 |
| **9.** | **J’ai eu mon bébé contre moi pour la première fois au moment où j’en ai eu envie** | 🞏 | 🞏 | 🞏 | 🞏 |
| **10.** | **Les premiers instants avec mon bébé correspondaient à ce que j’avais imaginé avant d’accoucher** | 🞏 | 🞏 | 🞏 | 🞏 |

# A ce jour

|  | | Tout à fait | En partie | Pas tellement | Pas du tout |
| --- | --- | --- | --- | --- | --- |
| **11.** | **Je suis fière de moi** | 🞏 | 🞏 | 🞏 | 🞏 |
| **12.** | **J’ai des regrets** | 🞏 | 🞏 | 🞏 | 🞏 |
| **13.** | **J’ai un sentiment d’échec** | 🞏 | 🞏 | 🞏 | 🞏 |
